# An unusual presentation of an intraosseous epidermoid cyst of the anterior maxilla: a case report

**DOI:** 10.1186/1752-1947-8-252

**Published:** 2014-07-14

**Authors:** Figen Atalay, Oyku Gulmez, Aylin Ozsancak Ugurlu

**Affiliations:** 1 Department of Hematology, Baskent University Istanbul Medical and Research Center, İstanbul, Turkey; 2 Department of Cardiology, Baskent University Istanbul Medical and Research Center, İstanbul, Turkey; 3 Department of Pulmonary Medicine, Baskent University Istanbul Medical and Research Center, İstanbul, Turkey

**Keywords:** Cardiotoxicity, Cyclophosphamide, Lymphoma therapy

## Abstract

**Introduction:**

Cardiac toxicity is one of the life-threatening complications of cancer therapy. Systemic anticancer treatments may exert their own toxic effects or can aggravate adverse effects of other drugs. We report a case of cyclophosphamide-induced cardiotoxicity in a patient with normal cardiac functions before chemotherapy.

**Case presentation:**

A 66-year-old Caucasian woman with a mediastinal mass diagnosed with Burkitt lymphoma underwent chemotherapy with rituximab-hyperfractionated-cyclophosphamide-vincristine-doxorubicin-dexamethasone. On the seventh day of chemotherapy, she developed dyspnea. An electrocardiogram demonstrated low voltage in the limb and precordial leads. It also showed diffusely increased myocardial echogenicity, mild pericardial and pleural effusion, generally impaired biventricular systolic functions with a left ventricular ejection fraction of 31%, and right ventricular mid-apical akinesia, even though she had normal biventricular functions before chemotherapy. Cyclophosphamide-induced cardiotoxicity was suspected and she was given treatment for congestive heart failure. Her dyspnea decreased and she was discharged on the tenth day with a left ventricular ejection fraction of 37% and normal right ventricular function. After 1 month, echocardiography showed normal biventricular functions with a left ventricular ejection fraction of 60%.

**Conclusions:**

Drug-induced cardiotoxicity, therefore, should be taken into consideration when using cyclophosphamide therapy, especially when anthracyclines are co-administered. Close communication between hematologists and cardiologists is required.

## Introduction

Cardiac toxicity is one of the life-threatening complications of cancer therapy. Systemic anticancer treatments may exert their own toxic effects or can aggravate the adverse effects of other drugs. Cyclophosphamide-induced cardiotoxicity (CIC) is a well-known adverse effect and not that uncommon [1]. Symptoms occur usually within 1 to 3 weeks, and although in some patients cardiotoxicity resolves without late consequences, the mortality rate can be as high as 43% [2-4]. Drug side effects are usually seen after a single administration. Previous and/or contaminant anthracycline treatment, previous radiation, age older than 50 years, and the presence of left ventricular (LV) dysfunction [1,4] are the risk factors of drug-induced cardiotoxicity. We report a case of CIC in a patient with normal cardiac functions before chemotherapy.

## Case presentation

A 66-year-old Caucasian woman with a history of hypertension was admitted to our hospital with a recent diagnosis of Burkitt lymphoma. Her lactate dehydrogenase level was high and the diameter of her mediastinal mass was more than 10cm. Her physical examination was normal, and basal electrocardiography (ECG) showed sinus rhythm with a heart rate of 72 beats/minute. Two-dimensional transthoracic echocardiography (TTE; Siemens, Acuson Sequoia, C512) revealed normal biventricular functions with an LV ejection fraction (LVEF) of 60%, mild mitral and tricuspid regurgitation, and moderate pericardial effusion.

A risk assessment of the patient put her into a high-risk category and she underwent rituximab-hyperfractionated-cyclophosphamide-vincristine-doxorubicin-dexamethasone (R-Hyper-CVAD) chemotherapy protocol. Her laboratory values are summarized in Table 1. She received high-dose cyclophosphamide 300mg/m^2^ twice daily for 3 days, doxorubicin 25mg/m^2^/day for 2 days, rituximab 375mg/m^2^/day for 1 day, dexamethasone 40mg/day for 4 days, and vincristine 2mg/day for 2 days. The total treatment dose of cyclophosphamide and doxorubicin received was 1800mg/m^2^ and 50mg/m^2^, respectively. She was given allopurinol 300mg/day perorally, sodium bicarbonate (8.4%, 10 flacon/day) infusion for 24 hours before chemotherapy, and mesna 600mg/m^2^/day for 2 days as prophylaxis against tumor lysis syndrome and hemorrhagic cystitis, respectively. She also received granisetron 2mg/day and lansoprazole 30mg/day as antiemetogenic and gastric prophylaxis, respectively.

**Table 1:** Patient’s laboratory values

	**First day of chemotherapy**	**Seventh day of chemotherapy**
**Hemoglobin, g/dL**	13	13.1
**White blood cell, μL**	5750	10,000
**Neutrophil, μL**	5160	6300
**Platelet, μL**	201,000	189,000
**AST, U/L**	24	26
**LDH, U/L**	535	329
**BUN, mg/dL**	10	12
**Creatinine, mg/dL**	0.42	0.37
**Calcium, mg/dL**	10	9.8
**Potassium, mmol/L**	4.4	4.5
**Uric acid, mg/dL**	3.5	3.2
**CRP, mg/dL**	2.8	71.6

Abbreviations: AST, aspartate transaminase; BUN, blood urea nitrogen; CRP, C-reactive protein; LDH, lactate dehydrogenase.

The patient developed dyspnea on the seventh day of therapy. A physical examination revealed blood pressure of 100/60mmHg and a heart rate of 110 beats/minute. On chest auscultation, no inspiratory sounds were heard at lower zones and inspiratory crackles were heard at middle zones. Neither cardiac murmurs nor S3 were heard. An ECG showed low voltage in the limb and precordial leads. TTE showed diffusely increased myocardial echogenicity, mild pericardial effusion, and generally impaired biventricular systolic functions with an LVEF 31% and right ventricular mid-apical akinesis. Manifest pleural effusion was also detected. Drug-induced cardiotoxicity (myocarditis) was suspected. Furosemide and ramipril were started. The beta-blocker therapy the patient was already taking for hypertension was continued. After 12 days, TTE showed an LVEF of 37% and normal right ventricular functions. Her dyspnea decreased and she was discharged on day 20. After 1 month, TTE showed normal biventricular functions with an LVEF of 60%.

After the first course of the R-Hyper-CVAD chemotherapy protocol, she underwent a high-dose methotrexate and cytarabine cycle. She had severe neutropenia and pneumonia. She had no cardiac failure symptoms during this chemotherapy course, but she declined another course of chemotherapy. She is still in remission despite the abbreviated course of chemotherapy.

## Discussion

Although progress has been made in therapeutic modalities of neoplastic diseases with a subsequent improvement in mortality and morbidity, life-threatening toxicities, especially cardiotoxicity, resulting from chemotherapy protocols remain a problem. Detection of cardiac injury after chemotherapy is crucial in order to start treatment as early as possible. Drug-related cardiac toxicity has been associated mostly with cyclophosphamide-containing regimens [4].

CIC usually occurs within 1 to 3 weeks, but no definite risk factors have yet been identified [5]; however, risk appears to be related to single dose rather than cumulative drug dose, older age, previous or concomitant use of anthracycline, and prior mediastinal irradiation [5]. The pathomechanism of CIC involves direct endocardial injury, followed by extravasation of toxic metabolites resulting in damage to cardiomyocytes, interstitial hemorrhage, and edema [1,4]. The incidence of fulminant congestive heart failure is reported to be 5% to 19% [4,6]. Symptoms may include arrhythmias, acute fulminant heart failure, myopericarditis, pericardial effusion, cardiac tamponade, and even death.

The incidence of anthracycline-induced cardiotoxicity (AIC) varies from 4% to 36% depending on cumulative dose for doxorubicin [5]. AIC has been categorized as acute, early-onset chronic progressive, and late-onset chronic progressive. It presents as dilated cardiomyopathy with a cardiac morbidity of 1% to 4% in the acute and subacute forms. Acute cardiotoxicity occurs during or shortly after the infusion with an incidence of <1%, and involves arrhythmias, heart failure, pericarditis-myocarditis syndrome, and nonspecific ST-T changes, which are usually reversible. Subacute cardiac toxicity occurs within a few weeks, clinically resembles myocarditis, and has an associated 60% mortality. Clinically, the most significant cardiac toxicity of the anthracyclines leading to LV dysfunction is late cardiac toxicity related to the cumulative anthracycline dose. Risk factors for anthracycline cardiotoxicity include cumulative dose; intravenous high single dose; elderly age; female gender; use of other concomitant agents such as cyclophosphamide, taxanes, or trastuzumab; prior mediastinal irradiation; and underlying cardiovascular disease [5].

Rituximab-related cardiotoxicity is usually in the form of arrhythmias, reported as 8% in patients treated for lymphoma. Interleukin-6, tumor necrosis factor-alpha, and other cytokines released after rituximab use may lead to cardiac arrhythmias, vasoconstriction, platelet activation, and/or rupture of atherosclerotic plaque [7,8]. In treatment of diffuse large B-cell lymphoma, addition of rituximab to cyclophosphamide-doxorubicin-vincristine-prednisolone (CHOP) chemotherapy regimen did not change the cardiotoxicity risk [9].

The only medical history of our patient was hypertension. She was receiving metoprolol as an antihypertensive treatment and no another medication. Her cardiac evaluation before chemotherapy was applied and there were no cardiac failure symptoms and findings in her physical examination, despite mild mitral and tricuspid regurgitation and moderate pericardial effusion. Her total cyclophosphamide dose was 56mg/kg for 3 days, which is not a high-dose cyclophosphamide regimen (120 to 200mg/kg) [2]. She also received anthracycline 50mg/m^2^, again within safe limits for cardiac toxicity. In addition, she received Hyper- CVAD chemotherapy protocol with rituximab. Because the total cumulative dose of anthracycline, the best predictor of AIC, she received is within normal limits and acute and subacute forms of AIC are rare, generally minor, and reversible, we ruled out the diagnosis of AIC. Because of the timing of cardiotoxicity and its relation to a single dose, she was diagnosed with CIC.

## Conclusions

Early detection and management of heart failure after a chemotherapy regimen is difficult. Cardiac functions should be assessed at baseline with TTE, even in asymptomatic patients, before starting combination therapy for cancer. Repeat assessments, during and after treatment, should also be considered. Cardiac monitoring with ECG and TTE is important in detecting clinical signs of cardiotoxicity. Drug-induced cardiotoxicity should be kept in mind while using cyclophosphamide therapy, especially when anthracyclines are coadministered. Close communication between hematologists and cardiologists is required.

## Consent

Written informed consent was obtained from the patient for publication of this case report and any accompanying images. A copy of the written consent is available for review by the Editor-in-Chief of this journal.

## Competing interests

The authors declare that they have no competing interests.

## Authors’ contributions

FA was the primary doctor of the patient, correspondence author, and made corrections in manuscript in light of reviewers’ comments. OG was the cardiology doctor of the patient pre- and post-treatment, provided cardiac evaluation of the patient, and wrote the manuscript draft. AOU diagnosed the lymphoma and made grammatical corrections. All authors read and approved the final manuscript.
